# Efficient spatio-temporal modeling for sign language recognition using CNN and RNN architectures

**DOI:** 10.3389/frai.2025.1630743

**Published:** 2025-08-25

**Authors:** Kasian Myagila, Devotha Godfrey Nyambo, Mussa Ally Dida

**Affiliations:** ^1^School of Computation and Communication Science and Engineering, The Nelson Mandela African Institution of Science and Technology, Arusha, Tanzania; ^2^Faculty of Science and Technology, Mzumbe University, Morogoro, Tanzania

**Keywords:** CNN-GRU, CNN-LSTM, deep learning, ELU activation function, sign language, Tanzania sign language

## Abstract

Computer vision has been identified as one of the solutions to bridge communication barriers between speech-impaired populations and those without impairment as most people are unaware of the sign language used by speech-impaired individuals. Numerous studies have been conducted to address this challenge. However, recognizing word signs, which are usually dynamic and involve more than one frame per sign, remains a challenge. This study used Tanzania Sign Language datasets collected using mobile phone selfie cameras to investigate the performance of deep learning algorithms that capture spatial and temporal relationships features of video frames. The study used CNN-LSTM and CNN-GRU architectures, where CNN-GRU with an ELU activation function is proposed to enhance learning efficiency and performance. The findings indicate that the proposed CNN-GRU model with ELU activation achieved an accuracy of 94%, compared to 93% for the standard CNN-GRU model and CNN-LSTM. In addition, the study evaluated performance of the proposed model in a signer-independent setting, where the results varied significantly across individual signers, with the highest accuracy reaching 66%. These results show that more effort is required to improve signer independence performance, including the challenges of hand dominance by optimizing spatial features.

## 1 Introduction

Globally, it is estimated that more than 5% of the world's population has a speech impairment challenge (Tripathi et al., 2023). This population usually uses sign language as a means of communication. Sign languages (SLs) make use of the “corporal-visual” channel, produced with body movement, and perceived with the eyes. This form of communication is different from what people without the challenge use (Adaloglou et al., 2021). Usually, human beings use a vocal-auditory channel to communicate. These two forms of communication have created a communication barrier between these two groups, since most of the population without the challenge lacks proficiency in sign language (Abdou, 2018). The barrier has severe impacts on the speech impaired population as they are left behind economically, socially, and politically (Das et al., 2023).

Sign language is the language conveyed through the movement of body parts, usually hands, fingers, head, and facial expression (El-Alfy and Luqman, 2022). Like vocal auditory languages, sign language develops spontaneously wherever this group gathers and communicates. In the world, it is estimated that there are more than 300 sign languages that are used by different societies (Kothadiya et al., 2023).

As sign language is visual-based, recognition of sign language will be helpful in removing the barrier between people without speech impairment and the impaired population. However, recognition or translation of signs is a very challenging problem as the task involves an interpretation between visual and linguistic information (Kothadiya et al., 2023). Therefore, the area demands more studies to understand the patterns in these different sign languages that exist in the world.

In recent years, the field of image and video understanding using computer has witnessed great advancement due to the ability to collect and process large volumes of data. Through this, several applications have been created with excellent performance in various visual tasks such as image classification, object detection, semantic segmentation, and action recognition. This success is greatly contributed by the use of deep learning algorithms (Ma et al., 2022).

Usually, sign language is categorized into finger spelling (Ligal and Baral, 2022) and word spelling (Ismail et al., 2022). Finger spelling maps the alphabets of the language with different orientations of the hand fingers. In contrast, word spelling usually involves the movement of body parts, including hands, fingers, heads, and sometimes facial expression. Based on this, finger spelling can be easily mapped to a single frame (Zhang et al., 2022), while word spelling may require more than one frame (Abdullahi and Chamnongthai, 2022). This behavior has contributed to the application of two different types of algorithms, those which do not consider sequential data and those capturing sequential data.

There are a number of techniques that can be employed in sign language recognition. However, recent performance of deep learning techniques on image and video recognition has attracted more studies involving sign language recognition, from the classical convolution neural network (CNN) (Fang et al., 2019) to the current state-of-the-art algorithm, which is the vision transformer (Kothadiya et al., 2023). Apart from CNN and vision transformers, there are several algorithms that have been used to solve the challenges encountered in the process of sign language recognition, including 3DCNN (Al-Hammadi et al., 2020), recurrent neural network (RNN) (Abdou, 2018), VGG16 (Kabisha et al., 2022), GoogleNet (Duwairi and Halloush, 2022), ResNet (Shin and Jung, 2023), long short-term memory (LSTM) (Luqman and Elalfy, 2022), and gated recurrent unit (GRU) (Boukdir et al., 2023).

These algorithms can be grouped into three categories, where the first is the CNN architecture derivatives that are commonly used in image recognition tasks, including 3DCNN, VGG16, GoogleNet, and ResNet (Aksoy et al., 2021). The other group is the neural network architecture designed to capture and process sequential data; this includes RNN, GRU, and LSTM (Nosouhian et al., 2021). The last is the vision transformer, which was primarily designed for natural language processing (NLP) tasks. Vision transformer treats parts of the images as sequences of patches, enabling it to leverage the transformer's capability for handling sequences (Khan et al., 2022). However, transformers are more demanding in computational resources (Tao et al., 2024).

Several approaches have been employed in the sign language recognition process, including the datasets used and the type of device used to capture images or videos. As some of the signs can be captured and easily recognized by a single frame, some studies have used images (Begum et al., 2023; Siddique et al., 2023). Video datasets are also used in some of the studies that map the number of frames to a single sign (Khader et al., 2019; Yu et al., 2022). The datasets have been collected using different devices, including webcams (Shamrat et al., 2021), 3D cameras (Mejía-Peréz et al., 2022), and mobile phone cameras (Kishore et al., 2018), and some studies integrated cameras and motion sensors (Abdullahi and Chamnongthai, 2022).

Moreover, there are several deep learning models that have shown better performance in recent studies in similar tasks (Chai et al., 2021). However, the temporal relationship remains a challenge in most activity recognition tasks (Beddiar et al., 2020). In the context of sign language recognition, ignoring temporal relationships would result in loss of critical information as the sequence of frames defines the meaning of a word.

Recent studies that focused on dynamic words have addressed the challenge by dealing with spatial-temporal characteristics of videos. In this approach, a number of techniques have been employed including hybrid architectures of CNN and RNN variants such as LSTM and GRU (Ihsan et al., 2024; Kumari and Anand, 2024; Noor et al., 2024; Shashidhar et al., 2024; Torun and Karacı, 2024). Moreover, the literature shows that LSTM has been the dominant approach in sign language recognition compared to the GRU model (Al Abdullah et al., 2024).

The CNN-LSTM and CNN-GRU architectures are hybrid deep learning models that combine CNN with the RNN model to process spatio-temporal data (Challa et al., 2022). In both models, CNN layers are used to extract spatial features from input data such as images, video frames, or multichannel time series. These extracted features are then passed through either LSTM units or GRUs to capture temporal dependencies. While LSTMs use a more complex gating mechanism (input, forget, and output gates) to manage long-term memory, GRUs employ a simpler structure with reset and update gates, resulting in fewer parameters and faster training. CNN-LSTM tends to perform better with long sequences due to its richer memory structure, whereas CNN-GRU is more computationally efficient and often performs similarly in tasks with shorter sequences (Alsulami et al., 2024). These architectures have been relevant for tasks such as video classification, human activity recognition, and time series forecasting, where modeling spatial and temporal patterns is important (Dua et al., 2023). Therefore, the study focuses on investigating the performance and learning efficiency a combined architectures of CNN and RNN variants (CNN-LSTM and CNN-GRU) in sign language recognition. In addition, the study proposes the use of activation functions that can further facilitate the convergence of the models.

This study uses Tanzania Sign Language (TSL) datasets collected using different mobile phones, with the aim of developing a deep learning model that can perform optimally despite of image qualities. TSL is used by the speech-impaired population in Tanzania. Since the common vocal language in Tanzania is Kiswahili, the TSL signs are usually mapped to the Kiswahili language. The study collected videos of word signs and used them to develop a model that can classify the signs into their respective meanings.

In addition, our study goes beyond traditional evaluations by investigating the performance of these architectures in a signer-independent setting, a well-recognized challenge in sign language recognition (Tao et al., 2024). Furthermore, we explore an often overlooked factor in sign language recognition: the influence of hand dominance (Arkushin et al., 2023; Watkins and Thompson, 2017), on model performance. This aspect has not been widely discussed in previous studies.

In summary, our significant contributions to this work are summarized as follows: We used TSL video data collected using mobile devices to train and evaluate the model's generalization under varied image conditions. TSL being among of low-resource language in the context of sign language research; we propose CNN-GRU architecture with ELU activation function to reduce gradient vanishing and enhancing learning efficiency; We develop model and evaluate the performance of the model in a signer-independent setting, simulating real-world scenarios where it must recognize signs from unseen users.

## 2 Methodology

### 2.1 Proposed model

The hybrid architecture that captures spatial and temporal features includes the combination convolutional neural network and long short-term memory (CNN-LSTM) (Luqman and Elalfy, 2022) and the combination convolutional neural network and gated recurrent units (CNN-GRU) (Dua et al., 2023). Both CNN-LSTM and CNN-GRU architectures have the input layer, visual feature extraction, sequence learning, and output layer (Tasdelen and Sen, 2021).

**Figure 1:**
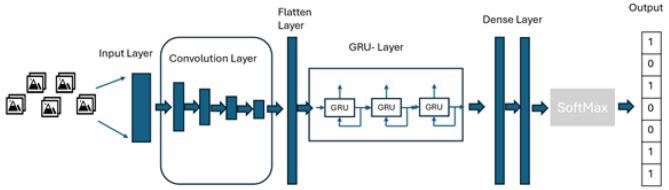
Proposed model architecture.

The study proposes the use of combined architecture as depicted in Figure 1. A similar architecture was also applied in the study by Luqman and Elalfy (2022) where they proposed the combined architecture of CNN and LSTM for Turkish sign language recognition. However, the study did not use CNN-GRU. The CNN-GRU architecture is proposed because GRUs often train faster and require fewer computational resources than LSTMs (Mateus et al., 2021). Moreover, the proposed architecture replaces the tanh activation function with the ELU activation function. The ELU activation function facilitates faster convergence by reducing the effect of vanishing gradient, which is one of the challenges in recurrent neural network architectures (An et al., 2022).

### 2.2 Datasets

The video datasets used in this study were created using recordings from five mobile phones: Google Pixel 4a, Google Pixel 3a, Samsung Galaxy A32, Samsung Galaxy A53, and Infinix Hot 12i. A custom-built mobile application was developed specifically for this task to guide signers through the signing process, ensuring consistency in the way signs were performed across sessions. The recordings were conducted in different settings to capture variability in lighting and background, with the aim of improving the generalizability of the dataset.

To promote diversity of datasets, the recordings involved 10 TSL signers. Each signer contributed 400 videos, one for each of the 40 selected sign words, resulting in a total of 16,000 videos. The 40 signs were selected based on a frequency analysis of public Kiswahili documents. Word frequency analysis was conducted using Python with libraries such as BeautifulSoup, Natural Language Toolkit (NLTK), and Pandas (Mishra and Sinha, 2022). The top 40 most frequently occurring words with existing TSL representations were selected, as presented in Table 1.

**Table 1:** TSL sign words, their translations in Kiswahili (a commonly spoken language in Tanzania), and their English equivalents.

**Code**	**Word (Kiswahili)**	**Translation (English)**	**Code**	**Word (Kiswahili)**	**Translation (English)**
0	mfano	Example	20	elimu	Education
1	kiwango	Extent	21	zana	Tools
2	jifunza	Learn	22	umuhimu	Importance
3	kazi	Work	23	stadi	Skills
4	hitaji	Need	24	soma	Read
5	jenga	Construct	25	sifa	Qualification
6	maalumu	Special	26	ulemavu	Disability
7	maana	Meaning	27	somo	Lesson
8	idadi	Quantity	28	shule	School
9	lingana	Match	29	Tanzania	Tanzania
10	mazingira	Environment	30	toa	Offer
11	hatua	Steps	31	mwalimu	Teacher
12	fundisha	Teach	32	shughuli	Activity
13	andaa	Prepare	33	mwanafunzi	Student
14	andika	Write	34	mtoto	Child
15	darasa	Class	35	mbili	Two
16	dhana	Concept	36	mbalimbali	Various
17	awali	Pre-school	37	sehemu	Part
18	aina	Type	38	njia	Path
19	eleza	Explain	39	michezo	Sports

Due to variability in video length due to signer articulation rate difference, frame rate, and gesture duration, pre-processing was performed to standardize the dataset for model input. The pre-processing pipeline followed a procedure adapted from (Ismail et al. 2022) which included frame extraction, sign segmentation, and frame normalization.

In frame extraction, each video was split into individual frames using OpenCV at the native frame rate. Sign segmentation involved isolation of only the relevant gesture portion, and frames captured before the start and after the end of the signing motion were removed. Frame normalization aimed at normalizing the sign sequence to 25 frames per sign. Signs with fewer frames were padded with interpolated frames, while longer sequences were uniformly down-sampled to retain the core gesture motion.

The frames were also resized to a fixed dimension of 256–256 pixels and pixel values normalized to the range [0, 1]. Each preprocessed video was treated as a batch, resulting in 16,000 batches, each containing a sequence of 25 frames representing a single sign. In general, the final datasets contain a total of 400,000 frames (16,000 videos – 25 frames each), standardized in both spatial and temporal dimensions to facilitate robust training and evaluation of dynamic sign recognition models.

### 2.3 Model development

The study was carried out on an Intel Core i7 (Base Clock 2.9GHz–5.0GHz Turbo Boost technology) processor with 32 GB RAM and 16 GB GPU. The Anaconda distribution package was used to enhance the platform setup and configuration. This package was used because it is an open source package (Al-Haija, 2022). Moreover, a Jupyter Lab notebook was used to undertake all the experiments and analysis using the Python programming language.

In model development, a holdout validation approach was used to ensure the model is not overfitting and the reliability of the model performance on the unseen data. The datasets were divided into a ratio of 80% for training, 10% for testing, and 10% for validation (Ko et al., 2019). The architecture is trained using back propagation (Wright et al., 2022) where the weight of every unit is adjusted based on the change in loss *L* similarly to the biases of unit which are as indicated on (1) and (4), where η is the learning rate. Cross-entropy *C* is used to regulate the optimization algorithm, which adjust weights using the chain rule. *C* is determined using (1) (Sarker, 2021).


(1) $$\begin{array}{l} C=\frac{1}{N}\overset{N}{\underset{i=1}{\sum }}L_{i} \end{array}$$

(2) $$\begin{array}{l} L_{i}=\overset{C}{\underset{j=1}{\sum }}y_{i,j}\cdot \log\left({ŷ}_{i,j}\right) \end{array}$$

(3) $$\begin{array}{lll} W^{l} & \leftarrow & W^{l}-\eta \cdot \frac{\partial L}{\partial W^{l}} \end{array}$$

(4) $$\begin{array}{lll} b^{l} & \leftarrow & b^{l}-\eta \cdot \frac{\partial L}{\partial b^{l}} \end{array}$$

### 2.4 Model evaluation

Four metrics, which are accuracy (Acc), precision (p), recall (r), and f1-score (f1), are used to assess the performance of the model. (Shin et al. 2023) also employed these metrics, with Acc serving as the primary metric to measure the overall accuracy of the developed model, (p) focusing on the rate of false positives, and r primarily focusing on the true positive rate. The f1 score recognizes the need for a balanced metric and accounts for both recall and precision. Since the model will be evaluated using the correctness of the output given input, Acc, p, r, and f1 are evaluated using True Positive (TP), True Negative (TN), False Positive (FP), and False Negative (FN) as indicated in (Equations 5–8).


(5) $$\begin{array}{l} \text{Acc}=\frac{TP+FP}{TP+FP+TN+FN} \end{array}$$

(6) $$\begin{array}{l} p=\frac{TP}{TP+FP} \end{array}$$

(7) $$\begin{array}{l} r=\frac{TP}{TP+FN} \end{array}$$

(8) $$\begin{array}{l} f_{1}=2\times \frac{p\times r}{p+r} \end{array}$$

## 3 Findings

### 3.1 Proposed model performance

**Figure 2:**
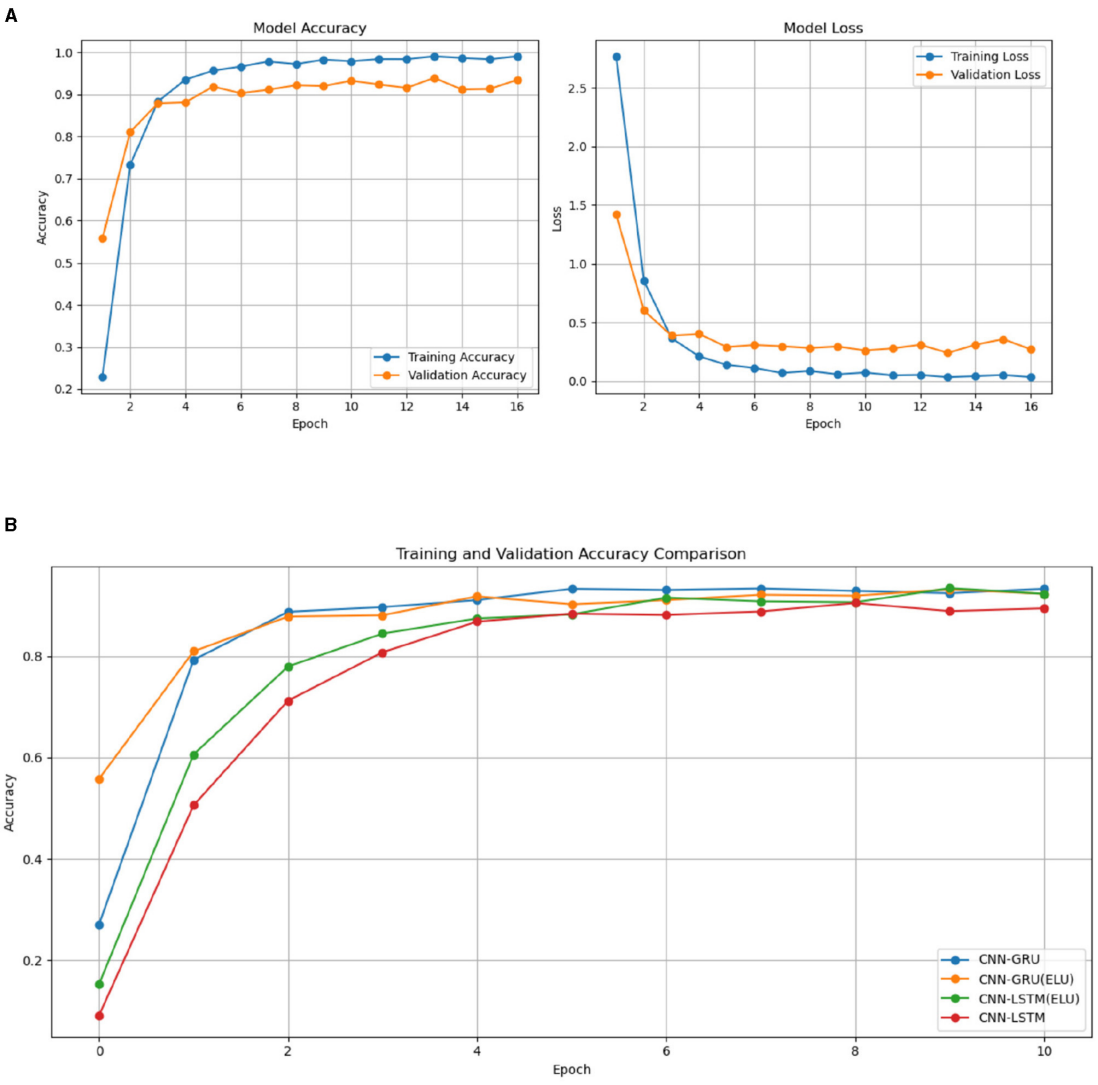
Training and validation graphs for accuracy and loss on CNN-GRU model training **(A)**. **(B)** Validation graphs for accuracy proposed model and related models.

Throughout the training and validation phases, accuracy and loss metrics were tracked and then plotted, as shown in Figure 2. To avoid over-fitting, an early stopping callback was applied, which halted the training process when no further improvement in validation performance was observed. This technique helped in maintaining model generalization without over training.

Figure 2 presents the results for validation accuracy form CNN-GRU, CNN-GRU (ELU), CNN-LSTM (ELU), and the CNN-LSTM model. The result demonstrate that the CNN-GRU model, utilizing the Exponential Linear Unit (ELU) activation function, demonstrated a higher convergence rate compared to the CNN-GRU architectures. This faster convergence suggests that the CNN-GRU model with ELU is better at adapting and stabilizing during training than the CNN-GRU model. Furthermore, the CNN-LSTM model with ELU outperformed the CNN-LSTM model with the tanh activation function, exhibiting a similar trend of improved convergence. These findings suggest that the ELU activation function contributes to faster and more stable convergence rates compared to the tanh function, underscoring its effectiveness in training deep learning models.

The developed models were validated and tested using a datasets comprising 20% of the entire datasets. The validation and testing results, detailed in Table 2, underscore the models' robustness and effectiveness. The CNN-LSTM model achieved a strong validation accuracy and testing accuracy of 92.4% and 93%, respectively. In particular, the CNN-GRU model, enhanced with the ELU activation function, outperformed slightly with a validation accuracy of 93.3% and a testing accuracy of 94% which is 1% higher than the CNN-LSTM model.

**Table 2:** Performance comparison of CNN-GRU models.

**Model**	**Accuracy (%)**	**Precision (%)**	**Recall (%)**	**F1 Score (%)**
CNN-LSTM	93	93	93	93
CNN-LSTM (ELU)	93	93	93	93
CNN-GRU	93	93	93	93
CNN-GRU (ELU)	94	94	94	94

These results reflect consistently high performance across all proposed evaluation metrics, indicating that the model not only accurately classifies instances (precision) but also captures a substantial proportion of relevant positive cases (recall). The balanced f1 score further confirms the alignment between precision and recall, highlighting reliability and robustness under various testing conditions. This uniformity across metrics demonstrates the model's well-rounded performance and its capability to maintain high accuracy while minimizing miss-classifications, underscoring its suitability for real-world applications.

**Figure 3:**
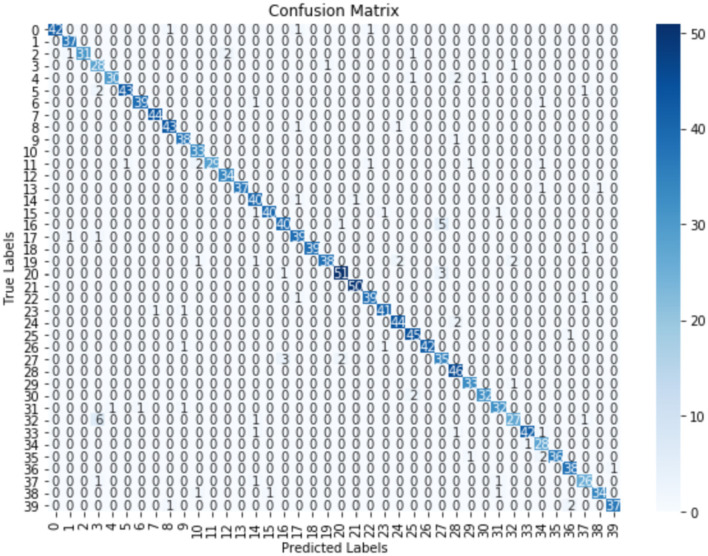
Confusion matrix for CNN-GRU with ELU activation function.

Furthermore, to gain deeper insight into the performance of the model, a confusion matrix was used. The plot reveals that most of the classes were accurately predicted, as shown by the prominent diagonal entries in Figure 3. This strong diagonal pattern underscores high precision of the model in classifying instances correctly into their respective categories, with minimal miss-classifications across classes.

### 3.2 Signer dependency

One characteristic of a good sign recognition system is being able to achieve good results on a signer who was not part of the training datasets. This is among the important factors that assess the generalizability of the model in classifying the sign (Calado et al., 2021). In this regard, the study used the TSL dataset and the proposed algorithms to assess their signer independence. The datasets were separated based on signer to achieve the same ratio. The model was trained using a dataset from 8 signers, which makes the similar ratio to the previous experiment of 8:1:1. However, the testing set is a dataset of an individual signer. The result of this task showed that the minimum accuracy was 25% and the maximum 66%, as indicated on Table 3.

**Table 3:** Signer-wise performance metrics.

**Signer**	**Accuracy (%)**	**Precision (%)**	**Recall (%)**	**F1 Score (%)**
Signer 1	51	61	51	48
Signer 2	66	71	66	63
Signer 3	52	58	52	48
Signer 4	42	53	42	40
Signer 5	25	26	26	22
Signer 6	45	57	45	45
Signer 7	54	61	54	55
Signer 8	64	72	64	62
Signer 9	50	54	50	46
Signer 10	47	55	47	48

**Figure 4:**
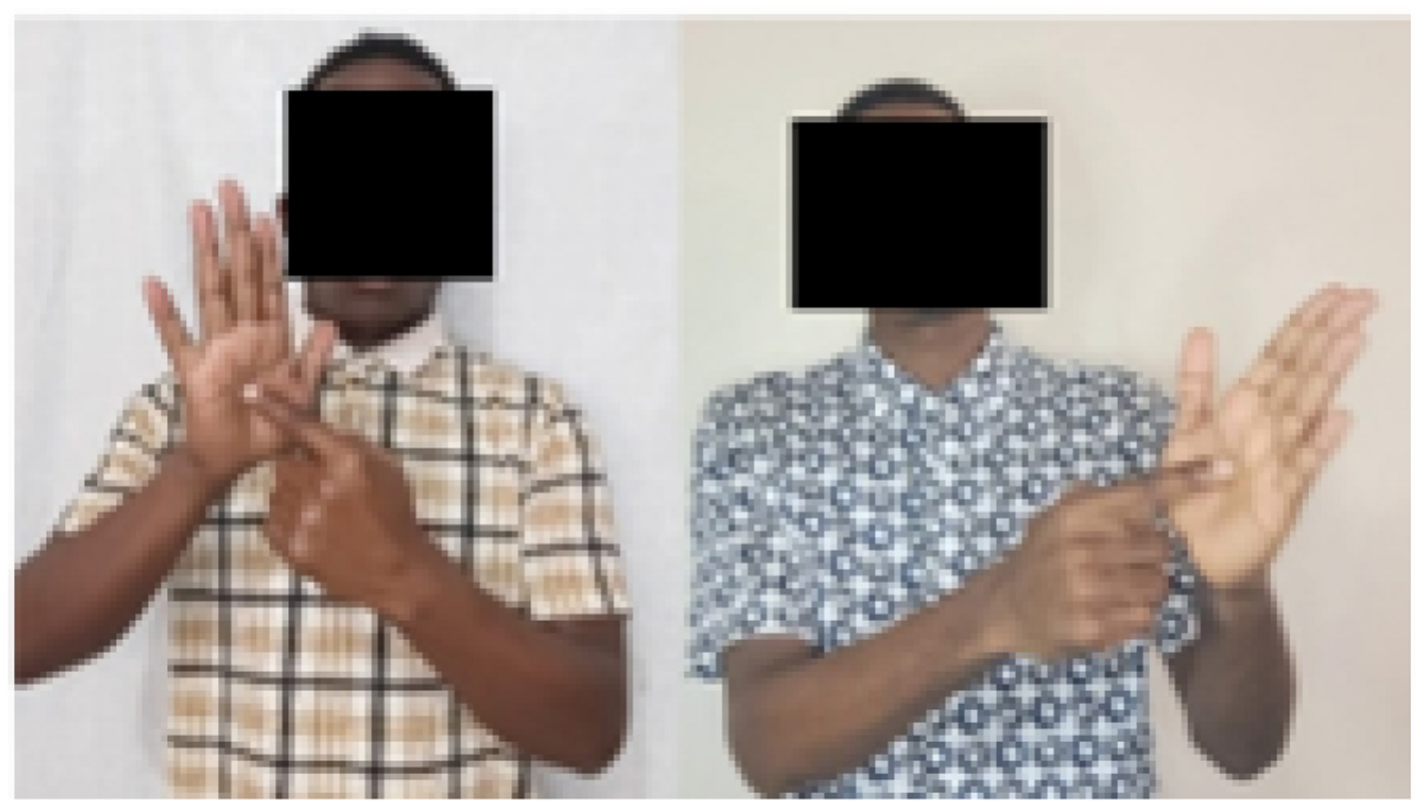
Left hand vs right hand orientation of signers.

Further investigation revealed that the minimum score was the result of signer hand orientation. Among the ten signers involved in this study, one is left-handed. This common human trait can potentially impact the model's performance, as a left-handed person would favor using the left-hand while a right-handed person would favor the right-hand. This difference can be observed in Figure 4 which are the frames extracted from the word “mfano”.

To highlight the impact of the left hand, we designed an experiment in which the testing and validation sets included a mix of two signers: one using the left hand and the other using the right hand. In the first case, the datasets were used as they are, and in the second case, the left-hand signer dataset was horizontally flipped. The accuracy of the model increased to 57% from 43%. Moreover, performance improvement was also observed on p, r, and f1, where they increased to 62%, 57%, and 56% from 51%, 43%, and 43%, respectively.

**Figure 5:**
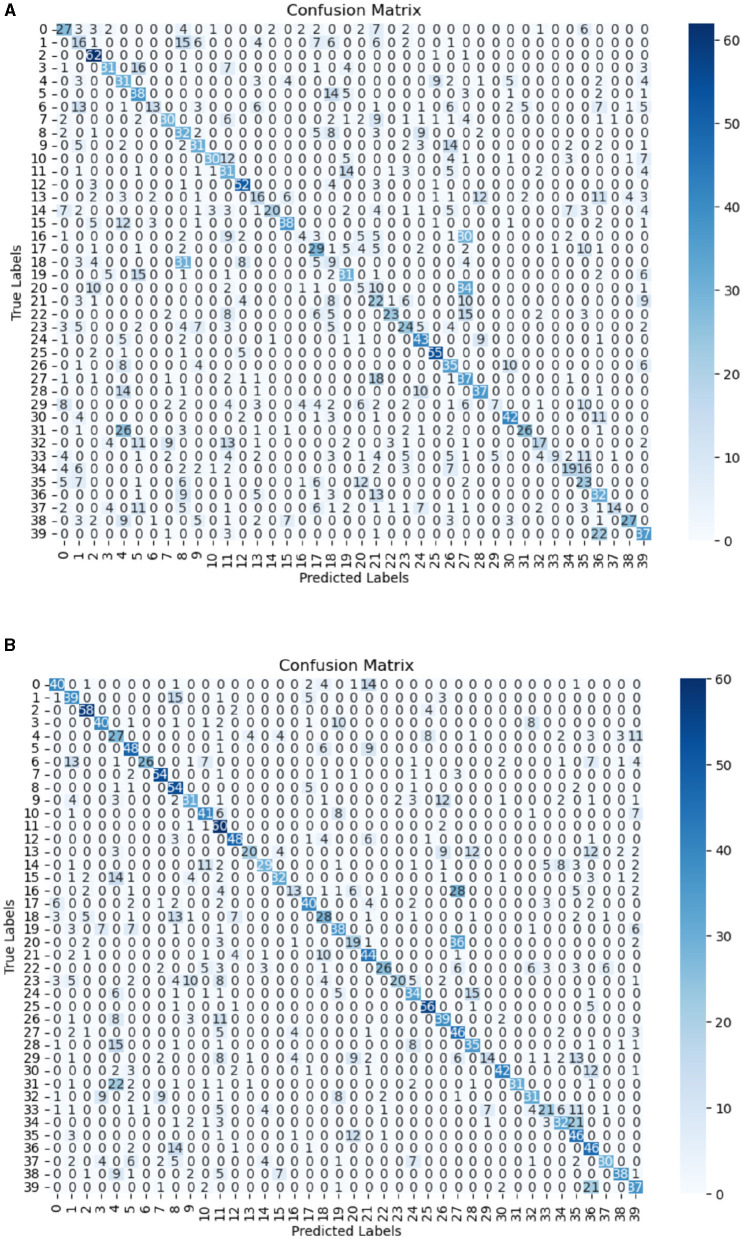
Confusion matrix for non-flipped datasets **(A)**. **(B)** Confusion Matrix for flipped datasets.

The confusion matrices for the task are presented in Figures 5A, B. On CNN-GRU with a flipped dataset, a significant increase in the number of correctly classified classes was observed in Figures 5A, B, as there is no class with less than ten correctly classified signs, which was not the case for the non-flipped dataset. The improvement can also be seen in the individual class. For example, for class with label 0, correctly classified classes increased from 27 to 40. This shows that the model can be significantly affected by the effect of hand dominance of the signer.

## 4 Discussion

Despite the fact that the datasets were collected in unconstrained environments and using various mobile phone devices, the proposed model demonstrates robust performance, achieving promising results compared to other similar studies. Table 4 provides a summary of related studies and their overall performance, where our model attains comparable or even superior results.

**Table 4:** Performance comparison of related studies.

**Study**	**Proposed model**	**Performance (%)**
(Al-Hammadi et al. 2020)	3DCNN	96.69
(Smith et al. 2021)	CNN	93.00
(Hussain et al. 2022)	MediaPipe+Random Forest	93.70
Luqman and Elalfy (2022)	CNN-LSTM	95.70
(Ihsan et al. 2024)	MobileNetV2-LSTM	95.83
(Kumari and Anand 2024)	MobileNet+LSTM+Attention	84.65
(Noor et al. 2024)	CNN-LSTM	94.40
(Chu et al. 2025)	CrossViViT	92.47
**Proposed Model**	**CNN-GRU**	**94**

Luqman and Elalfy (2022) employed a CNN-LSTM model, achieving an accuracy of 95.7% without using absolute frame differences. However, their model's performance significantly improved after incorporating absolute frame differences, highlighting the importance of feature manipulation techniques in improving model accuracy. This improvement suggests that, although our proposed CNN-GRU model already demonstrates strong performance, additional feature manipulation techniques could further boost its accuracy. Notably, our CNN-GRU model outperformed similar algorithms proposed by Luqman and El-Alfy, which was used as a reference model in this study.

Similarly, studies (Ihsan et al., 2024; Kumari and Anand, 2024; Noor et al., 2024) employed a hybrid architecture of CNN and LSTM to address the challenges of capturing spatiotemporal relationships and achieved comparable results. However, their studies did not include the GRU variant, which improves efficiency by reducing the number of gates, thereby lowering the number of trainable parameters. This enhancement improves training effectiveness and can be beneficial in reducing computational costs. (Al-Hammadi et al. 2020) used a 3DCNN model that achieved an accuracy of 87%. However, 3DCNN struggle to capture the sequential dynamics crucial for recognizing dynamic word signs, which involve a temporal component that 3DCNN are less suited to handle. This limitation is similar to the study by (Smith et al. 2021), who used a CNN focused on static gestures; also, this algorithm is lacking the temporal awareness required for dynamic gestures.

(Hussain et al. 2022) utilized pose estimation features alongside a Random Forest (RF) classifier. While this approach is beneficial for reducing the dimensionality of the input data to the sequential model, the proposed RF models do not capture the temporal relationships between frames. However, pose estimation techniques could be integrated with a sequence-based model to reduce the volume of information processed while preserving key gesture patterns, potentially improving model performance by focusing computational resources on essential motion characteristics.

In the signer-independent experiments, the proposed model demonstrated varying levels of accuracy across different signers. Table 5 summarizes the overall performance of signer-independent experiments across related studies. For example, (Al-Hammadi et al. 2020) achieved an accuracy of 34.9% in the Arabic Sign language dataset, yet their model's performance significantly varied on other datasets. In addition, (Aly and Aly 2020) achieved an accuracy of 20.5% without segmentation. However, the performance was significantly improved to 89.6% when the hand segmentation approach was used.

**Table 5:** Similar signer independent testing results.

**Study**	**Accuracy (%)**
(Aly and Aly 2020)	20.50
(Al-Hammadi et al. 2020)	34.9

Variation in signer-independent performance can be attributed to several factors, including inter- and intra-subject variability, changing illumination conditions, partial occlusions, differing points of view and resolutions, and background artifacts (Rastgoo et al., 2021). Given that the datasets were collected in unconstrained environments and using different devices, variability in performance was anticipated.

Furthermore, our study found that a signer's hand dominance can significantly impact model performance as hand dominance affects gesture dynamics and can introduce additional variability between signers. Models trained without accounting for hand dominance may struggle to generalize across different signers, leading to inconsistencies in recognition performance. This study finding highlights the importance of incorporating hand dominance as a factor in sign language recognition models, as doing so could improve robustness and adaptability, especially in signer-independent scenarios.

This study proposes the use of GRU over LSTM due to its simplified gating mechanism, which reduces the number of trainable parameters and improves computational efficiency without significantly compromising performance, as our study demonstrated. Notably, despite using a GRU-based model, our approach achieved high accuracy, outperforming the results reported by (Chu et al. 2025), where an accuracy of 92.47% was achieved for a 50-signs recognition task using a vision transformer (ViT) that aimed at reducing the computational demands. This highlights the effectiveness of proposed architecture in capturing spatio-temporal patterns, even without relying on more computationally demanding transformer-based models. This makes GRU particularly beneficial for real-time applications and development on limited computational resources. The study also proposes the use of the ELU activation function, as it helps mitigate the vanishing gradient problem and accelerates convergence, leading to more stable and efficient learning.

However, the hand dominance challenge highlighted by this study can be investigated further using other model including the ViT model. In addition, to ensure reliable performance in real-world applications, it is important to account for all relevant variations during dataset preparation and model development. This includes incorporating diverse signing styles, environmental conditions, and device types. Doing so will improve the model's robustness and inclusivity across a wide range of real-world scenarios.

## 5 Conclusion and future work

The work has presented the procedure and results of experiments on deep learning model performance in translating sign language signs using Tanzania Sign Language datasets collected using mobile phones. The study proposes the combined architecture of CNN and GRU, where ELU is used as an activation function. The proposed algorithm aimed to capture spatial features and temporal relationship of dynamic sign videos. In addition, the study investigates the performance of the developed model in the signer-independent mode. According to the study findings, the proposed model achieved an accuracy of 94%. Moreover, the finding on the signer independent experiment, where the model was developed using a datasets that was separated based on signer, showed significant differences that were expected due to variation of signers, and the scene where the signs were performed. Moreover, the left-hand orientation was also identified as one of the challenges for the revealed results, where the model performance increased by 14% when left-handed signer data were horizontally flipped. This will be taken for future works, where data augmentation techniques and skeleton-based features can be explored to address the challenges.

## Data Availability

The raw data supporting the conclusions of this article will be made available by the authors, without undue reservation.
